# Impact of conduit-filling interactions on the efficacy of fiber and hydrogel fillers in nerve conduits

**DOI:** 10.1016/j.isci.2025.113150

**Published:** 2025-07-18

**Authors:** Flavia Millesi, Sascha Mero, Sebastian Rihl, Sophie Steinwenter, Sarah Stadlmayr, Anton Borger, Paul Supper, Maximilian Haertinger, Leon Ploszczanski, Gerhard Sinn, Aida Naghilou, Lorenz Semmler, Christine Radtke

**Affiliations:** 1 Research Laboratory of the Department for Plastic, Reconstructive and Aesthetic Surgery, Medical University of Vienna, Spitalgasse 23, 1090 Vienna, Vienna, Austria; 2 Austrian Cluster for Tissue Regeneration, Vienna, Vienna, Austria; 3 Department of Urology, Medical University of Vienna, Währinger Gürtel 18-20, 1090 Vienna, , Vienna, Austria; 4 Department for Plastic, Reconstructive and Aesthetic Surgery, Medical University of Vienna, Währinger Gürtel 18-20, 1090 Vienna, Vienna, Austria; 5 Institute for Physics and Materials Science, University of Natural Resources and Life Sciences, Peter-Jordan-Straße 82, 1190 Vienna, Vienna, Austria; 6 Department of Physical Chemistry, University of Vienna, Währingerstraße 42, 1090 Vienna, Vienna, Austria

**Keywords:** Neuroscience, Materials science, Biomaterials

## Abstract

Nerve conduits offer an alternative to autologous nerve grafts, yet their clinical application remains restricted to short injuries with unsatisfactory outcomes. This study aimed to elucidate the factors responsible for these poor results. We systematically compared three commercially available conduits, assessing their impact on Schwann cells and fibroblasts *in vitro* alongside their material properties. Additionally, we evaluated the impact of luminal fillings —spider silk, hydrogel, and their combination— on cell behavior. All three hollow nerve conduits inhibited cell attachment, proliferation, and migration. Fillings significantly improved cellular responses, with effects varying depending on conduit type and material. Notably, spider silk and hydrogel influenced each other’s efficacy. Our experiments highlight the limitations of empty nerve conduits and served as a mechanistic exploration to unravel the reasons behind the superior outcomes observed with filled nerve conduits, underscoring the imperative for advancements in conduit design.

## Highlights

• FDA-approved nerve conduits inhibit Schwann cell attachment, proliferation, and migration
• Enhancements with hydrogel and spider silk fillings improved cell regenerative behaviors
• Spider silk fibers enhanced directed cell migration within the hydrogel filling
• Conduit morphology significantly influenced filling efficacy

## Graphical abstract

**Figure:**
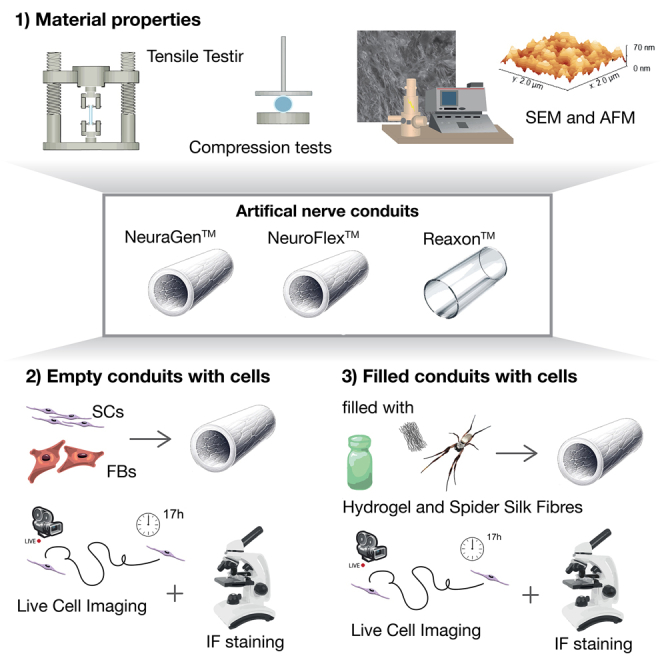

## Introduction

The intricate repair of peripheral nerve injuries (PNIs) stands as a persistent challenge in peripheral nerve regeneration (PNR). These injuries often lead to significant motor or sensory impairments, which profoundly impact the patients’ quality of life. Despite recent advances in nerve repair techniques, current reconstructive outcomes leave room for further improvement as regeneration is often constrained by the regenerative distance, causing muscle atrophy, fibrosis and functional deficits as well as neuropathic pain remains.^1^ Treatment approaches for nerve lesions vary based on the gap length.^1^ While significant progress has been made in treating short-gap PNIs, addressing long-distance injuries remains a considerable surgical challenge. The “critical” gap length, typically 3 cm in humans and 1.5 cm in rat models, correlates with substantially poorer outcomes beyond these thresholds.^2^ The gold standard for such critical injuries, commonly autologous sural nerve transplantation, presents drawbacks due to additional donor site morbidity from nerve harvesting, sensory loss, and limited nerve supply for repair in nerves with larger cross-sectional areas.^1^

To circumvent these limitations, researchers have been investigating artificial nerve guidance conduits (NGCs) as alternatives to nerve autografts.^3^ NGCs, which are hollow tubes inserted at the injury site, aim to bridge nerve defects and guide the regenerating axons toward the distal target. To allow an ideal environment for regeneration, NGC materials should shield regenerating axons, exhibit handling resistance and suturability as well as permeability for nutrient exchange, and provide a suitable framework for axon guidance.^4^ After conduit implantation, NGCs get encapsulated by a layer of fibrin, providing an essential scaffold for cellular ingrowth. However, in long-distance nerve defects, these fibrin cables degenerate before sufficient reinnervation can take place and only the hollow conduit remains.^5^ This presents a major issue in regenerative medicine, as no existing tissue-engineered conduit fully replicates a healthy nerve’s detailed structure and functions, and their successful application is currently confined to short and small-diameter nerve defects.^3^ Effective conduits should therefore not only be protective structures for regenerating nerves but also support cell ingrowth, prompting research into including specific microarchitectural features within the conduit lumen to aid regeneration.

Aiming to improve the microenvironment within the conduit, recent research has been enhancing hollow nerve conduits using luminal fillings such as hydrogels and fibers to make them more conducive for cell growth.^6^ Fibers within nerve conduits offer structural support and guidance for regenerating axons by promoting aligned axonal growth.^7^ Spider silk fibers have emerged as a promising material for filling conduits in PNR due to their remarkable biocompatibility, mechanical strength, and unique structural properties.^8^^,^^9^^,^^10^ These fibers provide an optimal scaffold for cell attachment, proliferation, and directed growth of nerve cells.^11^^,^^12^ Their high tensile strength and flexibility make them ideal for use in dynamic environments like peripheral nerves, which require both mechanical support and adaptability.^13^^,^^14^ Moreover, spider silk is biodegradable and elicits minimal immune response, reducing the risk of inflammation or rejection.^15^ In contrast, hydrogels have gained significant attention as a versatile material for PNR due to their biocompatibility, high water content, and tunable physical properties.^16^^,^^17^ These three-dimensional polymer networks closely resemble the natural extracellular matrix (ECM), providing a supportive and hydrated environment conducive to cell survival, proliferation, and differentiation.

The combination of spider silk fibers and hydrogels leverages the benefits of both materials, creating a composite scaffold that supports PNR more effectively than either component alone. Unfortunately, fundamental *in vitro* studies investigating conduits and fillings as a combination are missing and research only focuses on separate components. This renders understanding the interactions of cells on empty conduits as well as the interplay between conduits and filling impossible.

Therefore, this study systematically compared the NGCs NeuraGen, NeuroFlex, and Reaxon *in vitro*, assessing Schwann cell (SC) and fibroblast (FB) behaviors on both empty as well as filled nerve conduits. Our experiments revealed inhibitory effects of all three conduits on cell growth when used without fillings and significant improvement of cell behaviors after the introduction of spider silk and hydrogels as filling were observed. Interestingly, the degree of improvement was depended on conduit type and their respective material properties. Material characterization revealed distinct structural differences between chitosan and collagen conduits which correlated with the efficacy of the hydrogel filling. Our findings highlight why combining biomaterials remains a challenging task and demonstrate that the outcomes of material combinations need to be studied *in vitro* prior to *in vivo* application.

## Results

### NGCs inhibit Schwann cell and fibroblast elongation and proliferation

To understand the possible barriers of cell proliferation within the commercial conduits, we first investigated SCs and FBs behavior on the empty NGCs. Following PNIs, SCs exhibit substantial morphological changes during their transdifferentiation into a repair phenotype, characterized by enhanced cell elongation.^18^ The role of FBs is more controversial. While they excrete pro-regenerative growth factors and synthesize ECM after PNI, they have been known to induce scar formation. NGCs should regulate FB migration; allowing it, but keeping it lower than SC migration.

Seeing as we used GFP expressing cells to visualize the cells on the conduits, we first employed immunofluorescence stainings to ensure the expression of GFP and specific markers in transgenic SC and FB cultures (Figure S1). Both cell types showed adequate GFP expression and while SCs expressed the typical markers S100 and Sox10 besides NGFR, FBs were positive for PDGFR-a in addition to Thy1.

**Figure 1:**
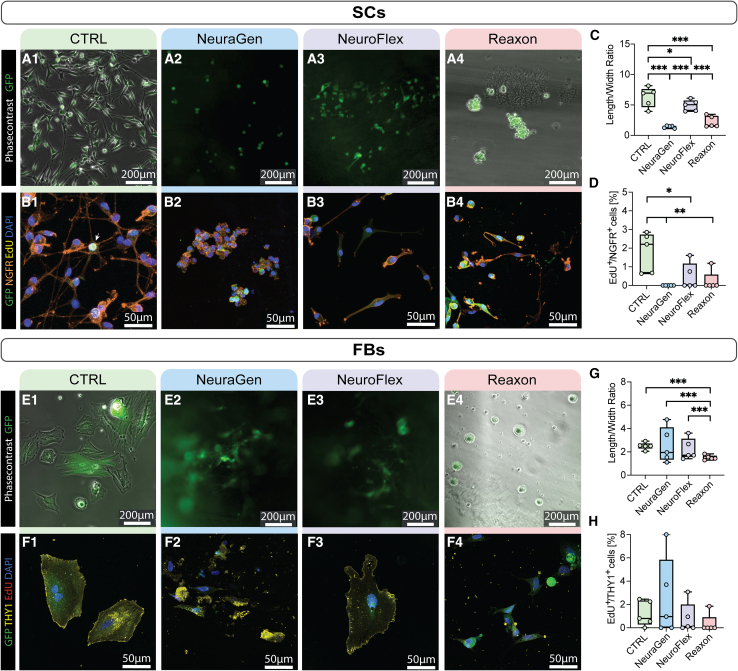
The three NGCs inhibit Schwann cell and fibroblast elongation and proliferation (A) Representative phase contrast and fluorescence micrographs of SCs on 1) uncoated wells (CTRL) and 2–4) on NGCs. Scale bar: 200μm. (B) Representative confocal micrographs of GFP^+^ SC cultures stained for NGFR, EdU, and DAPI. Scale bar: 50μm. (C) Boxplot depicting length/width ratio of SCs (*n* = 5). (D) Boxplot depicting percentage of EdU^+^/NGFR^+^ cells (proliferating SC) (*n* = 5). (E) Representative phase contrast and fluorescence micrographs of FBs. Scale bar: 200μm. (F) Representative confocal micrographs of GFP^+^ FB cultures stained for THY1, EdU and DAPI. Scale bar: 50μm. (G) Boxplot depicting length/width ratio of FBs (*n* = 5). (H) Boxplot depicting percentage of EdU^+^/THY1^+^ cells (proliferating FBs) (*n* = 5). Boxplots depict median and interquartile range. ∗ *p*-value <0.05, ∗∗ p-value <0.01, ∗∗∗ *p*-value <0.001, two-way ANOVA.

Upon seeding onto the NGCs, noticeable morphological changes were observed. In contrast to uncoated control conditions (CTRL) where SCs displayed a bi- and tripolar morphology with long, thin processes, SCs on the conduits exhibited fewer and shorter processes (Figure 1A). On NeuraGen, SCs showed no visible processes, while they had a few short processes on Reaxon (Figures 1B2 and 1B4). Reaxon is transparent compared to the collagen conduits, resulting in notably brighter images (Figures 1A4 and 1E4). On NeuroFlex, SCs displayed a morphology more comparable to elongated repair SCs (Figure 1B3). Quantitative analyses supported these observations. While SCs in CTRL exhibited multiple processes, with an average length/width ratio of 6.78, SCs on the NGCs showed significantly lower ratios (Figure 1C). Addition of EdU prior fixation allowed visualization of proliferating cells and demonstrated reduced proliferation of SCs on the NGCs compared to the uncoated wells on which SCs proliferated around 3%. NeuraGen showed no proliferating SCs (0%), while SCs on NeuroFlex and Reaxon proliferated 0.47 and 0.24%, respectively (Figure 1D).

FBs in CTRL displayed a planar and flat morphology with a length/width ratio of about 2.5, while FBs on the collagen-based NGCs appeared round and small. Reaxon exhibited limited FB attachment, resulting in a round morphology and reduced spreading of FBs (Figure 1E). Quantitative analyses revealed significant differences in the length/width ratio for FBs on Reaxon, indicating reduced elongation (Figure 1G). No significant differences were observed in the percentage of proliferating FBs across the conditions (Figure 1H).

In brief, all NGCs significantly reduced cell elongation and SC proliferation, with the highest elongation observed for SCs in NeuroFlex.

### NGCs markedly reduce SC migration, but not FB migration

Prolonged denervation causes neuromuscular junction degeneration and permanent loss of reinnervation capacity.^19^ In nerve gaps exceeding 3 cm, basal lamina and bands of Büngner degrade before regeneration completes, leading to SC atrophy and chronic denervation. Efficient SC migration to lesion sites is essential for functional recovery, and NGCs must support this process for successful axonal regeneration.

**Figure 2:**
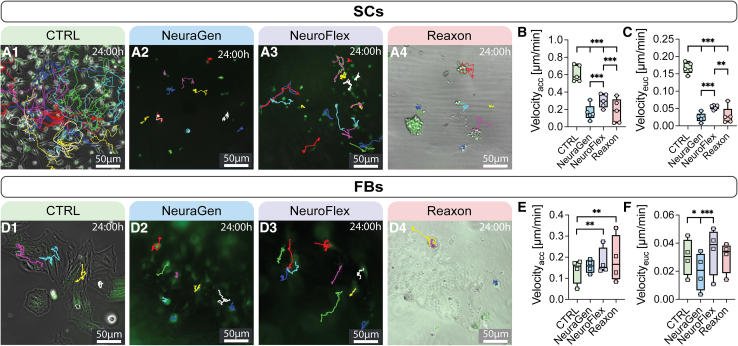
The three NGCs, especially NeuraGen and Reaxon, reduce cell migration (A) Magnified representative images of GFP^+^ SCs on (1) uncoated wells (CTRL) and (2–4) on NGCs after 24 h of live cell imaging. Colored lines represent SC migratory tracks. Scale bar: 50μm. (B) Boxplot depicting accumulated velocity (Velocity_acc_) of SCs in μm/min (*n* = 5). (C) Boxplot depicting Euclidean (effective) velocity of SCs in μm/min (*n* = 5). (D) Magnified representative images of GFP^+^ FBs after 24 h live cell imaging. Colored lines represent FB migratory track. Scale bar: 50μm. (E) Boxplot depicting accumulated velocity (Velocity_acc_) of FBs in μm/min (*n* = 5). (F) Boxplot depicting Euclidean (effective) velocity of FBs in μm/min (*n* = 5). Boxplots depict median and interquartile range. ∗ *p*-value <0.05, ∗∗ p-value <0.01, ∗∗∗ *p*-value <0.001, two-way ANOVA.

SCs and FBs were seeded on top of the NGCs and live cell imaging videos were recorded for 24 h. Figures 2A1–2A4 and D1–D4 show micrographs displaying cells and their tracked migration paths (colored lines) after 24h. SCs in CTRL group exhibited significantly faster migration on uncoated cell culture wells (CTRL) with about 0.61 μm/min compared to SCs on the three NGCs (0.16, 0.30 and 0.18 μm/min, respectively) (Figure 2B). For simplicity, this figure only includes the accumulated and effective (Euclidean) velocity of the cells. The effective velocity represents the directionality of the cells. We chose effective velocity and distance over directionality (effective/accumulated) as directionality depends on total distance covered and does not convey the true directness of a cell’s migratory path. The values for distance mirror the results of the velocity, and Table S1 provide the respective distance and directionality results. No significant difference was observed between the total velocity of SCs on NeuraGen and Reaxon (Figure 2B). However, SCs on NeuroFlex displayed higher speeds than those on the other two NGCs (Figure 2B). Furthermore, SCs on NGCs migrated significantly less effectively than the CTRL, particularly on NeuraGen and Reaxon (Figure 2C).

There was no significant difference observed between the total velocity of FBs in the CTRL group and those on the collagen NGC NeuraGen (Figure 2E). Conversely, FBs on NeuroFlex and Reaxon exhibited significantly faster migration compared to the CTRL (Figure 2E). Furthermore, FBs on NeuroFlex and the CTRL demonstrated more effective migration compared to those on NeuraGen (Figure 2F).

To summarize, the live cell imaging results indicated that all three NGCs restricted SC migration while NeuraGen and Reaxon promoted FB migration. However, NeuroFlex allowed faster SC migration compared to the other two conduits.

### Hydrogel and spider silk filling affect cell nuclei morphology and Schwann cell proliferation

*In vitro* experiments confirmed that hollow nerve conduits alone do not support cell attachment, proliferation, or migration effectively. Consequently, we aimed to investigate whether and how filling materials, such as hydrogel and fibers, could improve commercial NGCs by creating a better microenvironment for regeneration. Our previous research showed that hydrogels, in particular the basement membrane extract hydrogel Cultrex, and spider silk fibers enable cell attachment, proliferation, and migration.^11^^,^^12^^,^^17^ We hypothesized that a combination of fillings will further improve the performance of nerve conduits over conduits filled with matrix or fibers, respectively.

**Figure 3:**
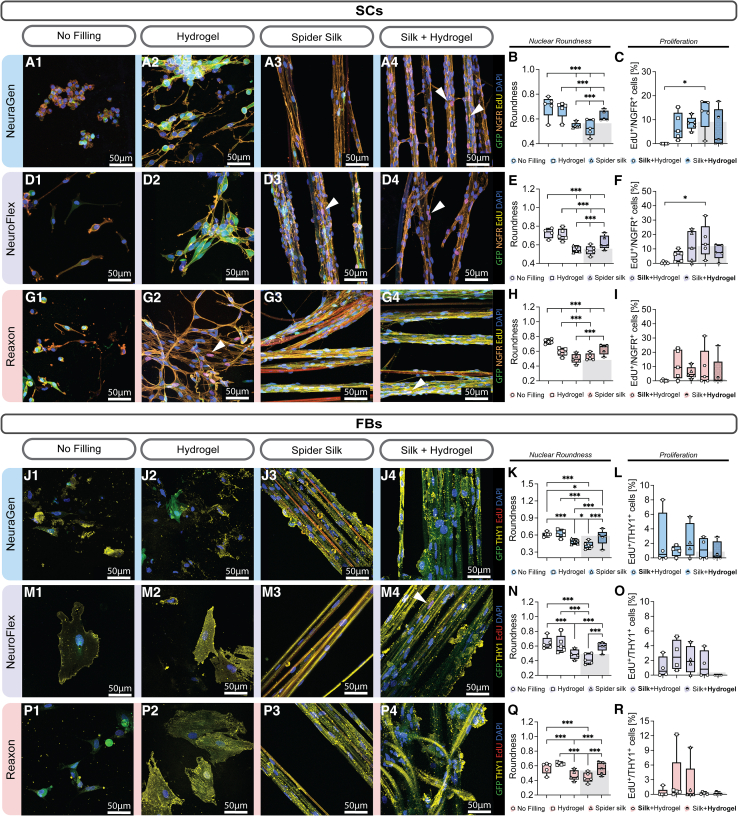
Hydrogel and spider silk fillings affect cell nuclei morphology and SC proliferation (A) Magnified representative confocal images of GFP^+^ SCs on NeuraGen stained for NGFR, EdU and DAPI. Scale bar: 50μm. (B) Boxplot depicting nuclear roundness of SCs on NeuraGen (*n* = 5). (C) Boxplot depicting percentage of proliferating SCs on NeuraGen (*n* = 5). (D) Magnified representative confocal images of SCs on NeuroFlex stained for NGFR, EdU and DAPI. Scale bar: 50μm. (E) Boxplot depicting nuclear roundness of SCs in μm^2^ on NeuroFlex (*n* = 5). (F) Boxplot depicting percentage of proliferating SCs on NeuroFlex (*n* = 5). (G) Magnified representative confocal images of SCs on Reaxon stained for NGFR, EdU and DAPI. Scale bar: 50μm. (H) Boxplot depicting nuclear roundness of SCs in μm^2^ on Reaxon (*n* = 5). (I) Boxplot depicting percentage of proliferating SCs on Reaxon (*n* = 5). (J) Magnified representative confocal images of GFP^+^ FBs on NeuraGen stained for THY1, EdU and DAPI. Scale bar: 50μm. (K) Boxplot depicting nuclear roundness of FBs on NeuraGen (*n* = 5). (L) Boxplot depicting percentage of proliferating FBs on NeuraGen (*n* = 4). (M) Magnified representative confocal images of FBs on NeuroFlex stained for THY1, EdU, and DAPI. Scale bar: 50μm. (N) Boxplot depicting nuclear roundness of FBs in μm^2^ on NeuroFlex (*n* = 5). (O) Boxplot depicting percentage of proliferating FBs on NeuroFlex (*n* = 4). (P) Magnified representative confocal images of FBs on Reaxon stained for THY1, EdU, and DAPI. Scale bar: 50μm. (Q) Boxplot depicting nuclear roundness of FBs in μm^2^ on Reaxon (*n* = 5). (R) Boxplot depicting percentage of proliferating FBs on Reaxon (*n* = 5). Arrowheads indicate proliferating cells. Boxplots depict median and interquartile range. Grey bars behind boxplots show combined mean of cells in filling combination. ∗ *p*-value <0.05, ∗∗ p-value <0.01, ∗∗∗ *p*-value <0.001, two-way ANOVA.

SCs and FBs were seeded on NGCs filled with spider silk, hydrogel as well as spider silk embedded in hydrogel. In the latter condition, we evaluated an equal number of cells on silk fibers surrounded by hydrogel (denoted as **Silk**+Hydrogel) and cells within the hydrogel surrounding the silk fibers (denoted as Silk+**Hydrogel**) to expose how the combination of biomaterials affects behavior of cells on the individual fillings. Their overall mean can be seen in form of the gray column. After seeding cells onto the filled conduits, we observed significantly improved cell attachment and spreading compared to empty ones (Figure 3). The SCs in the hydrogel filling showed enhanced process outgrowth, particularly evident in Reaxon (Figures 3A, 3D, and 3G2). Cell processes were difficult to differentiate from silk fibers, therefore nuclei roundness was analyzed.^17^ The less round the nuclei, the more elongated the cell. We observed that on all three conduits, cell nuclei were less round on the silk fibers compared to empty conduits (Figures 3B and 3E). Notably, SCs had significantly less round nuclei in the hydrogel filling on the chitosan conduit compared to the empty Reaxon (Figure 3H), while on both collagen conduits, there was no notable difference in nuclei roundness between empty conduits and those filled with hydrogel (Figures 3B and 3E). Similarly, while there was no difference between the SC nuclei roundness in the hydrogel and Silk+**Hydrogel** group on Reaxon (Figure 3H), the SCs on the collagen conduits exhibited less round nuclei within the hydrogel surrounding the silk fibers compared to the hydrogel only group (Figures 3B and 3E). The proliferation of SCs did not significantly differ between various fillings on Reaxon (Figure 3I). However, on NeuroFlex, SCs proliferated notably more on the silk fibers embedded in hydrogel (15.44%) compared to the empty conduit (0.47%) (Figure 3C).

FBs' nuclei were notably less round on the spider silk fibers and on the silk fibers surrounded by hydrogel (**Silk**+Hydrogel) compared to the FBs on the empty conduits, filled with hydrogel and in the Silk+**Hydrogel** group (Figures 3K, 3N, and 3Q). No significant difference was observed between the FB nuclei on the empty conduit and those in hydrogel (Figures 3K, 3N, and 3Q). However, the FBs within the hydrogel surrounding the silk fibers (Silk+**Hydrogel**) were significantly less round compared to the control on NeuraGen (Figure 3K). There was no notable variation in the proliferation rates of the FBs across the different fillings on all three conduits (Figures 3L, 3O, and 3R).

In summary, the presence of spider silk fibers led to an elongation of the cell nuclei across all three nerve conduits, both by itself and in combination with hydrogel. Interestingly, there seemed to be an influence of the silk fibers on the cells within the hydrogel (Silk+**Hydrogel**) as they were significantly less round when combining both fillings compared to the hydrogel only filling. This effect was only observed on the collagen conduits. Additionally, the combination of spider silk fibers and hydrogel notably enhanced SC proliferation on silk fibers (**Silk**+Hydrogel) on both collagen conduits. This indicates a synergistic effect of the three materials with the hydrogel enhancing proliferation on the silk fibers and the silk fibers promoting elongation within the hydrogel. This was also evaluated by comparing the means of the individual fillings with the overall mean of the combination of fillings (gray columns in Figure 3); The cells were more elongated and proliferated more in the combination of hydrogel and spider silk fibers compared to the hydrogel and silk fiber filling, respectively. This indicates that the fillings positively affect each other.

### Hydrogel and spider silk fillings within nerve conduits enhance Schwann cell migration

**Figure 4:**
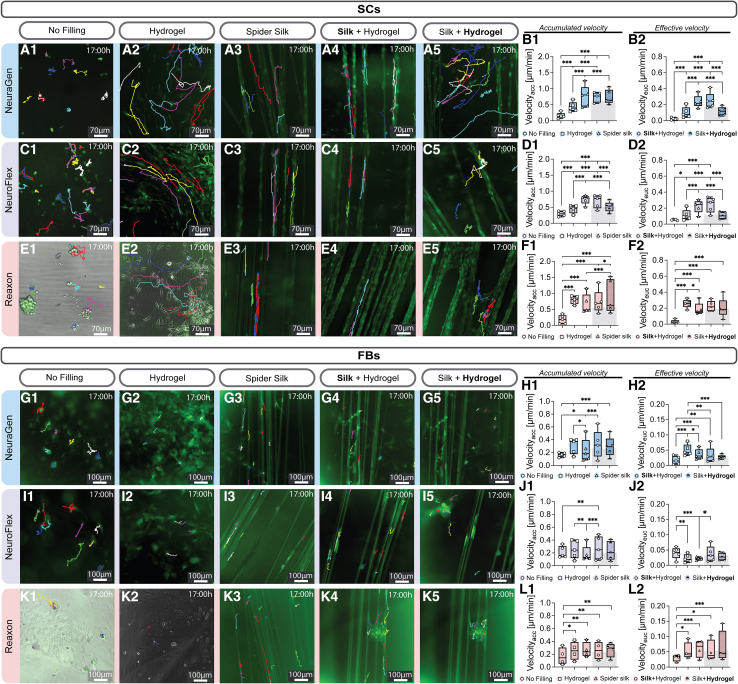
Hydrogel and spider silk fillings significantly enhance SC migration (A) Representative images of GFP^+^ SCs on NeuraGen after 17 h. Scale bar: 70μm. (B) Mean ± SD (1) accumulated velocity (Velocity_acc_) and (2) Euclidean (effective) velocity (Velocity_euc_) in μm/min of SCs on NeuraGen (*n* = 5). (C) Representative images of SCs on NeuroFlex. Scale bar: 70μm. (D) Boxplot depicting 1) accumulated velocity (Velocity_acc_) and 2) Euclidean (effective) velocity (Velocity_euc_) μm/min of SCs on NeuroFlex (*n* = 5). (E) Representative images of SCs on Reaxon. Scale bar: 70μm. (F) Boxplot depicting (1) accumulated velocity (Velocity_acc_) and (2) Euclidean (effective) velocity (Velocity_euc_) μm/min of SCs on Reaxon (*n* = 5). (G) Representative images of GFP^+^ FBs on NeuraGen. Scale bar: 100μm. (H) Boxplot depicting (1) accumulated velocity (Velocity_acc_) and (2) Euclidean (effective) velocity (Velocity_euc_) μm/min of FBs on NeuraGen (*n* = 5). (I) Representative images of FBs on NeuroFlex. Scale bar: 100μm. (J) Boxplot depicting (1) accumulated velocity (Velocity_acc_) and (2) Euclidean (effective) velocity (Velocity_euc_) μm/min of FBs on NeuroFlex (*n* = 5). (K) Representative images of FBs on Reaxon. Scale bar: 100μm. (L) Boxplot depicting (1) accumulated velocity (Velocity_acc_) and (2) Euclidean (effective) velocity (Velocity_euc_) μm/min of FBs on Reaxon (*n* = 4). Silk + Hydrogel shows the cells 4) on the spider silk surrounded by hydrogel and 5) in the hydrogel surrounded by the silk. Boxplots depict median and interquartile range. Grey bars behind boxplots show combined mean of cells in filling combination. ∗ p-value < 0.05, ∗∗ p- value < 0.01, ∗∗∗ p-value < 0.001, two-way ANOVA.

Given that quick SC infiltration into the nerve conduit is integral to regeneration, we next explored the influence of hydrogel and spider silk fillings on cell migration within the conduits. Figure 4 displays live cell imaging results, showcasing the migration of SCs and FBs on NGCs incorporating filling materials. The colored lines represent the migratory paths of each tracked cell (Figures 4A–4C and 4G–4K). For the combination of fibers and hydrogel, we again analyzed an equal number of cells on spider silk fibers (**Silk**+Hydrogel) and in the hydrogel (Silk+**Hydrogel**) for which representative images can be seen separately in Figure 4. Their overall mean can be seen in form of the gray column.

On all three nerve conduits, the fillings significantly enhanced SC migration, evident from the extended colored lines in the filled conduits compared to the empty ones (Figures 4A–4C). Notably, while the hydrogel significantly boosted SC migration compared to the empty conduits, the mean SC accumulation and effective velocities peaked on spider silk fibers, both alone and embedded in hydrogel within collagen conduits (Figures 4B and 4D). Conversely, on Reaxon, while all fillings increased SC accumulation and effective velocities significantly, SCs were overall fastest on the hydrogel only filling (Figure 4F). Examining the overall mean of the combination of hydrogel and spider silk fibers (in the form of the gray columns in Figure 4), there were no significant differences observed on Reaxon compared to the other fillings (Figure 4F). However, on collagen conduits, the combination significantly reduced the effective velocity of the SCs compared to those on silk fibers alone, but increased the effective velocity compared to SCs on hydrogel alone (Figures 4B and 4D2). This underscores the superior capability of spider silk fibers in facilitating directed migration within a three-dimensional matrix.

FBs exhibited a significantly higher velocity on all fillings on NeuraGen and Reaxon (Figures 4H and 4L), while on NeuroFlex, FBs were only significantly faster on the silk fibers surrounded by hydrogel compared to CTRL (Figure 4J1). On both collagen conduits, the FBs on the silk fibers surrounded by hydrogel (**Silk**+Hydrogel) were significantly faster than the FBs on the silk fibers alone (Figures 4H and 4J1). FBs migrated most effectively in the hydrogel on NeuraGen, but not on NeuroFlex where the fillings decreased the effective velocity compared to CTRL (Figures 4H and 4J2). There were no significant differences between the fillings on Reaxon (Figures 4L1 and 4L2).

In summary, all fillings significantly enhanced both accumulative and effective SC migration. The SCs’ speed was highest on the silk fibers on both collagen conduits while on Reaxon, it was highest in the hydrogel filling. Similarly, the fillings augmented FB migration, albeit observed only on NeuraGen and Reaxon, not on NeuroFlex.

### Conduit origin and composition influence filling efficacy

In our investigation of various NGC fillings, we observed differential efficacy across conduits, likely due to their distinct origins and morphologies. To address this, we further separated the combination of filling data and compared the conduits with one another for each filling.

First, we assessed nuclear morphology and cell proliferation rates across conduits with different fillings. While SC proliferation rates remained consistent across all conduits (Figures S2A2–S2D2), SCs exhibited significantly rounder nuclei on the two collagen conduits compared to Reaxon when in contact with hydrogel or silk fibers (Figures S2A1 and S2B1). Notably, there were no distinctions in SC nuclear roundness within the combination of fillings among the three conduits (Figures S2C1 and S2D1). Additionally, no notable variances were observed in FB nuclear roundness or proliferation rate across the conduits (Figures S2E1, S2H1, and S2H2).

**Figure 5:**
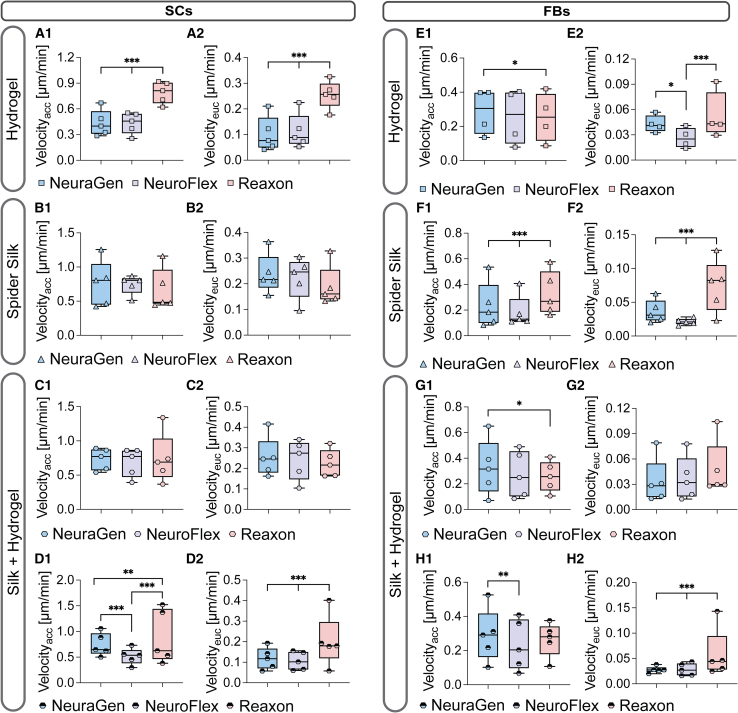
The two collagen conduits interact differently with the fillings than the chitosan conduit (A) Boxplot depicting (1) accumulated velocity (Velocity_acc_), and (2) Euclidean (effective) velocity (Velocity_euc_) of SCs in hydrogel (*n* = 5). (B) Boxplot depicting (1) accumulated velocity (Velocity_acc_), and (2) Euclidean (effective) velocity (Velocity_euc_) of SCs on silk fibers (*n* = 5). (C) Boxplot depicting (1) accumulated velocity (Velocity_acc_), and (2) Euclidean (effective) velocity (Velocity_euc_) of SCs on silk fibers surrounded by hydrogel (*n* = 5). (D) Boxplot depicting (1) accumulated velocity (Velocity_acc_), and (2) Euclidean (effective) velocity (Velocity_euc_) of SCs in hydrogel around silk fibers (*n* = 5). (E) Boxplot depicting (1) accumulated velocity (Velocity_acc_), and (2) Euclidean (effective) velocity (Velocity_euc_) of FBs in hydrogel (n = 4–5). (F) Boxplot depicting (1) accumulated velocity (Velocity_acc_), and (2) Euclidean (effective) velocity (Velocity_euc_) of FBs on silk fibers (n = 4–5). (G) Boxplot depicting (1) accumulated velocity (Velocity_acc_), and (2) Euclidean (effective) velocity (Velocity_euc_) of FBs on silk fibers surrounding hydrogel (n = 4–5). (H) Boxplot depicting (1) accumulated velocity (Velocity_acc_), and (2) Euclidean (effective) velocity (Velocity_euc_) of FBs in hydrogel around silk fibers (n = 4–5). Boxplots depict median and interquartile range. ∗ *p*-value <0.05, ∗∗ p-value <0.01, ∗∗∗ *p*-value <0.001, two-way ANOVA.

**Figure 6:**
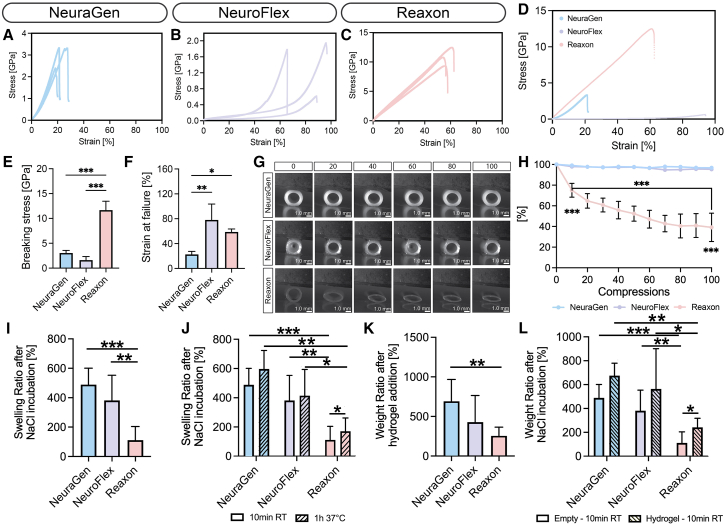
Collagen conduits show superior flexibility and liquid retention compared to brittle chitosan-based Reaxon® (A–C) Three replicate stress/strain curves of the three NGCs. (D) Mean stress/strain curves of the three NGCs in relation to each other. (E) Mean breaking stress in GPa for the three NGCs. (F) Mean maximum strain at failure in percent of the three NGCs. (G) Optical micrographs of conduits after every 10th compression. (H) Mean ± SD changes in conduit area in percent over the 100 compressions. Reaxon differs significantly from the other two conduits from the first until the last compression. (I) Mean swelling ratios of the dry conduits after 10 min in NaCl show significantly lower values for Reaxon compared to NeuraGen and NeuroFlex. (J) After 1 h incubation in NaCl at 37 °C, swelling ratios of Reaxon increased, while the collagen-based conduits remained stable. (K) Mean weight ratios after addition of hydrogel. (L) Mean weight ratios of hydrogel-filled conduits after 10 min in NaCl compared to the weight ratios of empty conduits after 10 min NaCl incubation. Bar plots depict mean ± SD. ∗ *p*-value <0.05, ∗∗ p-value <0.01, ∗∗∗ *p*-value <0.001, two-way ANOVA and paired t-test (*n* = 3).

We further delved into the influence of conduit type on cell migration in various fillings. Figure 6 compares the accumulated and effective velocity of SCs and FBs on the three respective nerve conduits for each filling. Table S1 provide the respective distance and directionality results. Notably, SCs on spider silk fibers displayed no significant differences in migration across conduits (Figures 5B, 5C1, and 5C2). However, SCs exhibited significantly faster migration – both accumulated and effective—within the hydrogel filling on Reaxon compared to the collagen conduits (Figures 5A1 and 5A2). SCs also demonstrated notably higher migration rates within the hydrogel surrounding silk fibers (Silk+**Hydrogel**) on NeuraGen compared to NeuroFlex, with Reaxon displaying the fastest migration (Figures 6D1 and 6D2).

For FBs, migration was notably faster on hydrogel-filled NeuraGen compared to Reaxon, yet they migrated significantly more effective on Reaxon than on NeuraGen, where they were also notably faster than on NeuroFlex (Figures 5E1 and 5E2). Additionally, FBs displayed significantly faster migration on spider silk fibers within Reaxon compared to collagen conduits (Figures 5F1 and 5F2). For the combination of fillings, FBs exhibited significantly slower migration on the silk fibers surrounded by hydrogel on Reaxon compared to NeuraGen, but no notable difference in effective velocity (Figures 5G1 and 5G2). Furthermore, within the hydrogel surrounding silk fibers, FBs migrated notably slower on NeuroFlex compared to NeuraGen and covered notably more distance effectively on Reaxon compared to both collagen conduits (Figures 5H1 and 5H2).

Evidently, the hydrogel filling had a more pronounced effect on SC and FB migration on Reaxon compared to NeuraGen and NeuroFlex, possibly attributed to the material properties between the conduits. This underscores the necessity of understanding conduit/filling interactions based on their synergistic effects.

### Collagen conduits show superior flexibility and liquid retention compared to brittle chitosan-based Reaxon

After revealing that the fillings’ efficacy differs between the chitosan conduit and the two collagen conduits, we aimed at identifying correlations to material properties as the possible underlying reason. First, tensile tests were employed to test the NGCs’ mechanical properties to assess their resilience to deformation. Deploying conduits in flexible anatomical sites, such as the wrist require them to maintain structural integrity under varying degrees of compression and tension that mimic the dynamic physiological environment.^20^

Tensile tests were conducted to assess the mechanical strength and flexibility of NGCs. The collagen-based NeuraGen exhibited an average breaking stress of approximately 3.3 GPa at a strain of 20% (Figure 6A). NeuroFlex, also collagen-based, demonstrated an average stress of 0.6 GPa and extending up to 100% before failure (Figure 6B). The chitosan-based Reaxon displayed the highest strength, averaging around 12 GPa, and endured a breaking strain of 60% (Figure 6C). A comparison of the conduits' representative performance is depicted in Figure 6D. Reaxon showcased the highest breaking stress, while NeuroFlex exhibited the lowest strength (Figure 6E). However, NeuroFlex displayed the highest flexibility, nearly doubling in length before failure, while NeuraGen was the least flexible, withstanding strains up to approximately 27% (Figure 6F).

Subsequently, compression tests were conducted on the NGCs. Following 100 compressions, the inner cross-sectional area changes were quantified (Figures 6G and 6H). While the collagen-based NGCs displayed similar behavior and did not show any changes, Reaxon revealed a brittle nature and an average loss exceeding 40% of its original area after 100 compressions (Figure 6H).

In addition to mechanical properties, we assessed the swelling behavior of the conduits, both in their native form and after hydrogel filling, to evaluate how each material interacts with physiological fluids and hydrogel matrices. After 10 min in 0.9% (w/v) sodium chloride solution (NaCl), both collagen-based conduits, NeuraGen and NeuroFlex, showed high and comparable swelling ratios (around 80%), while Reaxon displayed significantly lower swelling (around 20%), indicating limited fluid uptake (Figure 6I). Following an additional 1-h incubation at 37°C, Reaxon exhibited a significant increase in swelling, suggesting delayed fluid absorption under physiological conditions (Figure 6J). However, it remained significantly lower compared to NeuraGen and NeuroFlex, which reached equilibrium already within 10 min.

To evaluate hydrogel–conduit compatibility as well as to elucidate if the hydrogel remains in the conduit, hydrogel was pipetted on top of the conduits and weight measurements were taken to quantify hydrogel retention (Figure 6K). All three conduits showed similar weight gain after hydrogel addition although NeuraGen showed a significant higher weight ratio compared to Reaxon. To further assess how the hydrogel affects swelling behavior in liquid and to verify that the hydrogel stays within the NGCs even after submerging in fluid, the conduits + hydrogel were incubated for 10 min in NaCl and the weight change was compared to the conduits without hydrogel after NaCl incubation. In Reaxon, weight ratios significantly increased after hydrogel addition compared to without hydrogel (Figure 6L), suggesting that the hydrogel substantially contributed to the measured mass. In contrast, after exposure to NaCl, the weight ratios of the hydrogel-filled collagen conduits were comparable to the values observed in the empty conduits (Figure 6L). This observation may indicate that the collagen conduits either became saturated with the hydrogel and could not retain additional fluid, or that the hydrogel was no longer present, potentially due to partial dissolution of the hydrogel or replacement by NaCl.

### The chitosan conduit exhibited a smooth surface in contrast to the collagen conduits’ fibrous nature

Next, scanning electron (SEM) and atomic force microscopy (AFM) were utilized to discern surface disparities among the conduits. Not only does the efficacy of NGCs heavily rely on their luminal wall structure as for example lower porosity and denser wall structure of NGCs impede FB penetration and fibrous tissue infiltration, but higher porosity facilitates vascularization, fluid exchange, and nutrient diffusion possible contributing to the conduit’s interaction with a hydrogel filling.^21^

**Figure 7:**
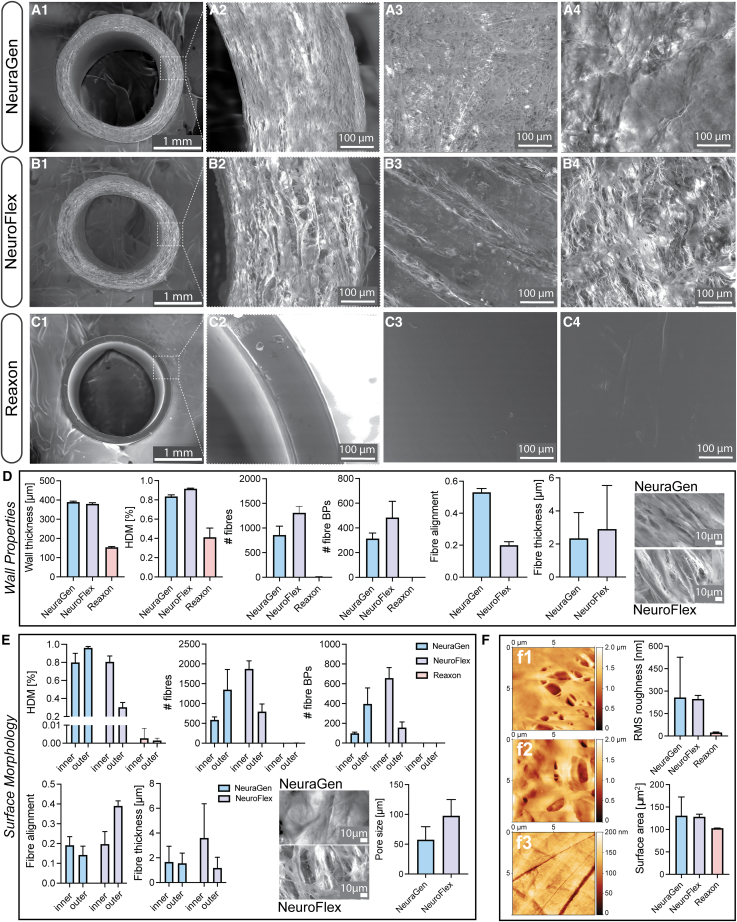
Reaxon exhibits a smooth surface in contrast to the collagen conduits’ fibrous nature (A–C) (1) Representative SEM images of NGCs and (2) magnifications of the wall structure and thickness as well as the (3) outer and (4) inner surface. Scale bar: 1000 and 100μm. (D) Wall properties including mean ± SD wall thickness, percentage of high-density matrix (HDM), the number of fibers, the number of branching points (BPs), fiber alignment, fiber thickness and pore size and representative magnifications of NeuraGen’s and NeuroFlex’s wall. Scale bar: 10μm. (E) Surface properties including mean percentage of HDM, the number of fibers, the number of BPs, fiber alignment, fiber thickness and pore size and representative magnifications of NeuraGen’s and NeuroFlex’s inner surface. Scale bar: 10μm. (F) AFM micrographs of (1) NeuraGen’s, (2) NeuroFlex’s, and (3) Reaxon’s inner surface. Mean ± SD inner surface root-mean-square (RMS) roughness in nm and inner surface area in μm^2^ for the three NGCs. Images of four respective positions for conduit wall, inner and outer surface were quantified per conduit. No statistical analyses have been performed as values are technical replicates. Bar plots depict mean ± SD.

SEM imaging highlighted distinct features between NeuraGen, NeuroFlex, and Reaxon conduits. NeuraGen and NeuroFlex exhibited tightly woven collagen fibers in their walls (Figures 7A1 and 7B1) depicted with higher magnification in Figures 7A2 and 7B2. Conversely, Reaxon demonstrated a notably smoother and more homogeneous wall structure (Figure 7C1), as well as an overall thinner wall (∼150 μm compared to ∼400 μm for the other conduits) (Figure 7C2). Quantifications confirmed these findings and revealed a lower fiber and branching point (BP) number but more aligned fibers in the wall of NeuraGen compared to NeuroFlex (Figure 7D). Since no fibers were present in Reaxon, no fiber characteristics were analyzed. No statistical analyses have been performed as values count as technical replicates.

Further inspection of the conduit surfaces revealed that while Reaxon displayed similar inner and outer surfaces (Figures 7C3 and 7C4), the collagen conduits exhibited differences between each other and their outer and inner surfaces (Figures 7A3, 7A4, 7B3, and 7B4). The inner surfaces of NeuraGen had less fibers and BPs compared to their outer counterparts. In contrast, it was the other way around for NeuroFlex (Figure 7E). Moreover, NeuroFlex demonstrated higher fiber alignment in the outer surface, but increased fiber thickness in the inner surface (Figure 7E). Lastly, NeuroFlex revealed to have bigger pores (around 100 μm) in the inner surface compared to NeuraGen (around 50 μm) which indicates less densely compacted collagen fibers in NeuroFlex. AFM imaging corroborated these findings and quantitative analysis from AFM data highlighted the chitosan conduit’s significantly smoother surface and reduced surface area in contrast to the collagen conduits but did not find any visible differences between the fibrous conduits (Figure 7F).

This comparative assessment offered crucial insights into the structural variations among NGCs, especially of different origins (collagen versus chitosan), potentially influencing their functional suitability in nerve regeneration.

### Interactions between conduit material properties and fillings shape cellular responses in NGCs

Understanding the interaction between cellular behaviors and material properties in NGCs is essential for optimizing conduit performance for specific applications. By examining not only the intrinsic material properties and cellular responses but also how different fillings impact these factors, we can gain insights into how material characteristics influence cell properties and how fillings can modify or enhance conduit performance.

**Figure 8:**
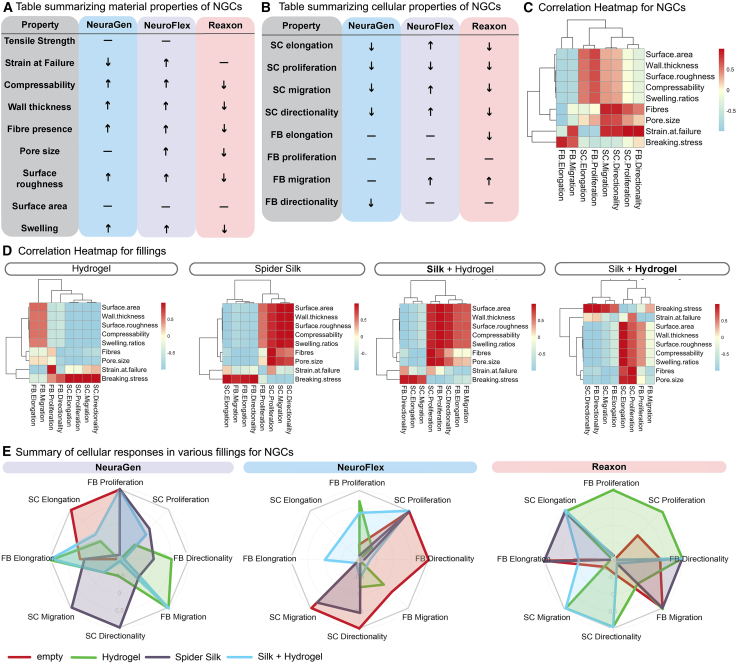
Summary of material and cellular properties of nerve guidance conduits (NGCs) and correlation analysis for different filling types (A) Table summarizing the material properties of three commercially available NGCs. Up and down arrows indicate higher and lower values, respectively, relative to the other conduits, while dashes represent moderate values. (B) Table summarizing the cellular responses in each NGC. Arrows indicate enhanced or reduced cellular responses in each conduit. (C) Correlation heatmap showing the relationship between material properties and cellular properties across all three NGCs. Red indicates positive correlations, blue indicates negative correlations, and white indicates weak or no correlations. (D) Correlation heatmaps for each filling type illustrating how different fillings influence the correlation between material properties and cellular responses. (E) Spider charts for each conduit showing how the fillings changed the conduit’s effect on cellular response. The center indicates −1 which means the conduit has the lowest value for that behavior among the conduits for the same filling, 0 means the conduit is average and 1, the outermost point, means the conduit has the highest value.

For better comparability, Figure 8A summarizes key material properties; Reaxon demonstrated the highest tensile strength, while NeuroFlex was the most flexible with the highest strain at failure. Reaxon also had a much thinner wall, lacked pores and fibers, and had a smooth surface and lower swelling ratios compared to NeuraGen and NeuroFlex. In contrast, both collagen-based conduits exhibited fibrous and porous structures, with NeuroFlex having pores approximately twice the size of those in NeuraGen. Figure 8B illustrates the cellular responses within each conduit. NeuroFlex promoted better elongation and migration of SCs, while Reaxon inhibited FBs elongation but promoted FB speed without increasing their effective migration.

Correlating these results revealed significant correlations between FB elongation and tensile strength, indicating that more rigid materials may better support FB elongation (Figure 8C). SC migration and directionality (in the form of effective velocity) showed high correlations with porosity, fiber presence, and strain at failure, suggesting that inner surface characteristics play an essential role in facilitating SC migration and directionality (Figure 8C).

We next visualized how different fillings alter the correlations between cellular responses and material properties (Figure 8D). In the Hydrogel-filled conduits, SC properties (proliferation, migration, and directionality) displayed strong positive correlations with tensile strength, while inner surface properties had a negative effect on these properties. In the spider silk-filled conduits, SC proliferation, migration, and directionality were positively influenced by inner surface properties (such as fiber presence and roughness), as well as compressibility, swelling and wall thickness. FB migration, elongation, directionality, and SC elongation also displayed strong correlations with breaking strength. On silk fibers within hydrogel (**Silk**+Hydrogel), FB migration and elongation were positively correlated with compressibility, swelling and wall thickness, while SC migration, in contrast to the silk fibers alone, was negatively correlated to inner surface properties. Conversely, in the hydrogel surrounding silk fibers (Silk+**Hydrogel**), increased SC elongation and proliferation went hand in hand with increased inner surface properties, while most other properties increased with tensile strength.

The presence of different fillings altered cellular responses within each conduit (Figure 8E). On NeuraGen, hydrogel significantly enhanced FB migration, while spider silk alone increased the migratory properties of SCs, but reduced SC elongation. The **Silk**+Hydrogel combination improved FB properties, but reduced SC migration and proliferation. On NeuroFlex, the addition of fillings generally decreased cellular response performance compared to the other conduits. Reaxon exhibited the most pronounced improvements with the addition of hydrogel, enhancing SC migration, proliferation, directionality, elongation, and FB proliferation and directionality. Spider silk increased FB migration, directionality, and FB and SC elongation, while the Silk+**Hydrogel** combination optimized SC elongation as well as SC migration and directionality, making Reaxon highly effective across these cellular metrics compared to the other conduits.

In summary, these results indicate that both intrinsic material properties and the choice of filling have substantial effects on cellular behaviors. Fillings could either alter the material characteristics of the conduit, such as by enhancing surface properties or providing additional rigidity or flexibility. In turn, material properties might significantly influence the effectiveness of different fillings. This nuanced analysis underscores the importance of selecting conduit materials and fillings that complement each other to optimize cellular behaviors for nerve regeneration applications.

## Discussion

Regardless of major advances over the last decades, the effective repair of nerve defects, where primary nerve repair is not possible, remains a notable challenge in current medicine.^1^ While the autograft remains the gold standard for bridging nerve gaps, its use is accompanied by drawbacks such as prolonged surgery, secondary incisions, and donor site morbidity.^1^ The emergence of synthetic NGCs promised a new frontier, yet only a handful of conduits (NeuraGen, NeuroMatrix, NeuroFlex, Neurolac, and Reaxon) have received FDA approval.^22^ Their application is recommended for short-nerve gaps under 3.0 cm.^2^ However, a recent systematic review and meta-analysis revealed that, even for short nerve lesions, they result in significantly less sensory recovery in contrast to nerve autograft and nerve allograft.^23^ Additionally, following surgery using an NGC, 26% of these patients experienced altered sensibility, 26% required revision surgery, and approximately 40% experienced pain.^23^ Despite those known limitations, the reason behind their lack of success remains elusive and systematic comparisons of synthetic nerve conduits and their effect on cells crucial for nerve regeneration *in vitro* are lacking or insufficient.

Our exploration involving three FDA-approved NGCs offers fresh insights into why they fail to repair extensive nerve deficits and their suboptimal efficacy for smaller injuries below 3.0 cm. NeuraGen and NeuroMatrix are quite similar which is why we chose to include only NeuraGen in our study. Neurolac had not yet received European approval at the start of these experiments which is why we refrained from incorporating it into this project.

NGCs’ primary goal is to act as a barrier to adjacent tissue during the regenerative process and fibrin which coats these conduits *in vivo* allows SC to infiltrate the conduit.^5^ However, it has been known to degrade before the regeneration process is complete. The rate of fibrin breakdown can vary significantly depending on factors such as the local environment, the presence of enzymes like plasmin, and the physiological conditions of the injury site.^24^ In cases of longer nerve gaps, the fibrin scaffold tends to degrade faster than the nerve can fully regenerate.^25^ This mismatch in timelines between fibrin degradation and complete PNR underscores the need for developing NGCs that support cell migration. In contrast, the three tested conduits inhibited SC behavior while having no adverse effect on FB proliferation and migration. The role of FBs in nerve regeneration is controversial seeing as they help to stabilize the injury site by forming a scar that can physically protect regenerating axons and provide a scaffold for cell migration but can also be an excessive barrier that blocks regenerating axons from reaching their target tissues.^26^ NGCs should therefore allow moderate FB adhesion and migration. In contrast, given their pivotal role in guiding regenerating axons, SCs are indispensable for successful PNR^18^ and NGCs should not only allow but particularly promote attachment and migration of these cells. Unlike the poor cell attachment we observed, another study described good cell distribution of ADSCs and SCs on NeuraGen by SEM after 48 h of cell seeding.^27^ However, SEM does not show if cells are viable. Moreover, while viability is the prerequisite for any cellular interaction, exploration of migration and proliferation would be more functionally significant.

The regeneration potential of PNIs is further highly influenced by the interplay between SC migration, axon sprouting and muscle atrophy. Our findings indicate that SCs migrate at an average rate of 0.37 mm per day on the empty nerve conduits (0.24 mm on NeuraGen, 0.55 mm on NeuroFlex and 0.30 mm on Reaxon). This is significantly slower than the reported axon regrowth rate of 1.0–3.0 mm/day.^28^ While SC migration does not directly translate to neurite outgrowth, successful neurite outgrowth depends on the prior presence of SCs which infiltrate the conduit from both the proximal and distal side of the injury.^24^ Considering studies by Gordon et al. which show that after denervation, muscles lose 50% of their weight by 100 days^29^ as well as a marked decline in muscle reinnervation potential after prolonged denervation,^19^ the maximal length of injury to employ an empty commercial NGC should be carefully evaluated. Delaying the repair of a tibial nerve defect in a rat model beyond one month led to a sharp decrease in muscle mass and motor function recovery^30^ which would account for a maximum working distance of 1.11 cm for the conduits tested in our experiments. This is below the critical length of 3.0 cm in human and 1.5 cm in rats.^23^

Materials like collagen, due to their inherent compatibility and regenerative properties, ease the approval process for medical devices.^3^ However, collagen’s role in the ECM is multifaceted; Type I collagen can be found in the epineurium, perineurium and endoneurium of a peripheral nerve and due to its supportive properties for PNR is commonly used in NGCs such as also in NeuraGen and NeuroFlex.^31^ However, it has been noted that SCs embedded in collagen I may not extend processes effectively and instead may adopt a spherical morphology indicating limited interaction with the matrix.^32^ Collagen type IV, a component of the basement membrane, has been shown to promote SC attachment, spreading and proliferation as well as enhance axonal outgrowth.^31^ Additionally, SCs rely on type IV collagen for assembling fibronectin in the matrix. The specific interaction between collagen types and cells can direct the regenerative process, either facilitating or impeding nerve repair.^31^ This underscores the importance of selecting the appropriate collagen type in the design of nerve conduits to ensure the promotion of beneficial cell behavior conducive to healing.^3^

Despite disappointing cell behavior results for all three NGCs, NeuroFlex displayed marginally superior performance, exhibiting favorable material properties as well as promoting cell spreading and migration compared to the others.

Another avenue for NGC improvement involves integrating channels, scaffolds, and matrices like hydrogels and fibers.^6^ In earlier experiments, we have identified a suitable hydrogel for PNR; Cultrex 3D Cell Culture Matrix, which promoted an elongated SC morphology and directed cell migration.^17^ Furthermore, we have previously shown that spider silk fibers allow cell attachment, proliferation, and migration making them the ideal longitudinal filling.^11^ We investigated whether Cultrex as well as dragline spider silk could improve the three NGCs *in vitro*. Furthermore, we explored whether combining the hydrogel with the silk fibers will result in improved cell properties. NeuraGen and Reaxon have already been applied in animal models in conjunction with a hyaluronic acid/laminin hydrogel which resulted in improved regeneration over the empty conduit but still inferior results to the autograft.^33^ In a rat sciatic nerve gap model, a NeuraGen conduit filled with fibrin-agarose hydrogel significantly improved clinical, functional, electromyographic, and histological outcomes, according to a study by Carriel et al..^34^ Ronchi et al. used skeletal muscle fibers and a Reaxon nerve guide to span a 10 mm median nerve gap in a clinical setting and demonstrated increased Neuregulin synthesis, a marker of SC viability.^35^ Indeed, our results indicate a significant improvement in cell attachment and migration on the filled nerve conduits. While few cells attached to the empty conduits, we noticed an increased cell number and improved cell spreading on conduits enhanced with spider silk. SCs on spider silk fibers in combination with hydrogel as a filler also showed increased proliferation rates compared to the empty collagen conduits. Each filling in any variation significantly improved SC migration, both in their total migration path and also regarding the distance the cells covered effectively.

Lastly, while the hydrogel substantially enhanced SC migration compared to all empty conduits, its impact was notably less pronounced on the collagen-based conduits compared to the chitosan-based Reaxon. This observed disparity in the effectiveness of the hydrogel filling across different conduit types presents an intriguing facet of our study. It hints at a potential interaction between the hydrogel and conduit material. To understand this phenomenon, we performed detailed material characterization of the investigated conduits. Given NGCs' potential use in flexible areas like the wrist, flexibility and mechanical robustness are critical.^20^ Tensile and compression tests on the conduits demonstrated NeuroFlex as the most flexible, and Reaxon as the toughest NGC. In contrast, NeuraGen showed limited flexibility and toughness. Notably, both collagen conduits maintained their original shape after compression, while Reaxon exhibited significant compression, suggesting a brittle nature. Several prior studies have emphasized the significance of conduit flexibility and mechanical robustness in enabling the optimal regenerative environment for nerve repair.^36^ While our study’s findings corroborate the importance of these mechanical features, they also highlight the variability among commercially available NGCs in terms of their mechanical characteristics. NeuroFlex’s superior flexibility aligns with studies emphasizing the benefits of flexible conduits in reducing compression-induced stresses, potentially enhancing the regenerative microenvironment.^36^ Moreover, our observations regarding Reaxon’s toughness yet brittleness resonate with studies discussing that hard materials, while tough, are less responsive to plastic deformations, which may result in brittle fracture due to crack initiation and/or propagation.^37^ Chitosan is generally known to be a brittle material in dry state,^38^ and while we did immerse the conduit in liquid for 10 min as recommended by the manufacturer, it might have been necessary to soak it for longer.

To further assess how material differences affect fluid interaction and matrix integration, we evaluated the conduits’ swelling behavior with and without hydrogel. After 10 min in 0.9% (w/v) sodium chloride (NaCl) solution, both collagen-based conduits (NeuraGen and NeuroFlex) showed high swelling ratios while Reaxon remained low. This is likely related to Reaxon’s smooth, non-porous surface, which may restrict fluid uptake.^39^ However, Reaxon’s swelling increased substantially after 1 h, suggesting delayed fluid uptake. These differences in swelling and retention have critical implications for surgical application and regenerative success. Conduits that swell excessively can become too soft or bulky, making precise suturing difficult and increasing the risk of material deformation or collapse during placement.^40^ This may impair alignment with the nerve stumps or introduce tension at the repair site. Conversely, delayed or incomplete swelling, such as observed in Reaxon, can result in a stiff, brittle conduit at the time of implantation, increasing the risk of poor sealing at the coaptation site or conduit fracture.^21^ In both cases, suboptimal swelling behavior compromises the surgeon’s ability to handle the conduit reliably and achieve complete enclosing around the nerve ends, which is essential to minimize gaps and promote guided regeneration.^41^ To elucidate if the hydrogel stays within or diffuses out of the conduit, we weighed the conduits before and after adding hydrogel. All conduits showed a comparable initial increase in weight, including Reaxon, suggesting that the hydrogel was successfully retained. Unlike passive liquid absorption, which relies on matrix porosity and hydrophilicity,^42^ the hydrogel may coat the inner surface or lodge within the lumen, elevating the weight ratio. Upon subsequent incubation in liquid, Reaxon still exhibited a higher weight compared to its value after liquid incubation without hydrogel, implying that a portion of the hydrogel remained in or adhered to the conduit rather than being absorbed or lost. In contrast, the collagen-based conduits did not show this significant weight increase following incubation, indicating that either they became saturated and could not retain additional fluid or that the hydrogel was gradually replaced. Both scenarios are suboptimal for nerve regeneration, as hydrogel displacement or lack of retention compromises its role in providing a supportive matrix within the conduit lumen.^39^

Recent advancements in NGC development have focused on modifying conduit structure and surface properties.^6^ This includes incorporating grooved or porous surfaces to enhance nutrient exchange, although high porosity has shown to promote fibrous tissue penetration.^21^ Our SEM and AFM analyses revealed that collagen-based conduits, NeuraGen and NeuroFlex, displayed porous, fibrous surfaces due to collagen fibers, whereas Reaxon exhibited a smoother, homogeneous surface, which likely contributed to differential cell attachment and migration. The importance of a porous and fibrous matrix for SC migration has been highlighted in recent studies,^17^ possibly explaining the slight improvement in SC migration in NeuroFlex compared to NeuraGen, as NeuroFlex consists of more fibers and larger pores. These findings suggest that NeuroFlex’s less densely packed collagen fibers may provide a more conducive environment for SC migration. Additionally, the higher porosity of the collagen-based conduits may facilitate quicker absorption and diffusion of hydrogel fillings as indicated by our swelling results, possibly diminishing their sustained effects on cellular behaviors compared to smoother, less porous conduits like Reaxon. This is supported by findings that observed that the benefits of hydrogel-filled conduits *in vivo* are more pronounced in non-porous environments, where hydrogel retention and cell-matrix interactions are optimized.^39^ However, a study by Huang et al. comparing NeuraGen and Reaxon filled with a hyaluronic acid/laminin hydrogel for a 15 mm rat sciatic nerve defect did not report significant differences between the conduits.^33^ One possible reason behind this could be different hydrogel properties; Cultrex forms a more cohesive, adhesive matrix that may interact differently with conduit porosity and composition, particularly favoring retention in less porous, smoother chitosan conduits. In contrast, the lower structural integrity of the hyaluronic acid/laminin hydrogel may have led to its uniform diffusion across conduit types *in vivo*, masking any material-specific differences.^43^ This highlights that not only the conduit material but also the choice of hydrogel strongly influences the effectiveness of NGC designs.

The correlation analysis further underscores the importance of matching conduit material properties with filling types. For example, high tensile strength and rigidity were found to diminish hydrogel efficacy, while silk fiber and Silk+Hydrogel combinations performed well in conjunction with these properties. The combined filling of hydrogel and silk fibers leverages the unique strengths of each component: the hydrogel provides a hydrated matrix that promotes cell attachment and initial proliferation, while the silk fibers add structural integrity, enhancing cell elongation and migration by offering an anchored scaffold. This synergy is particularly effective in conduits with larger pore sizes, such as NeuroFlex, where the addition of silk fibers in the hydrogel provides a structural scaffold within the porous environment, compensating for the hydrogel’s diffusion. A study using gellan-xanthan hydrogel conduits found that nanofibers within a hydrogel increased matrix resilience and lowered its deformability.^44^ Similarly, the silk fibers could have increase hydrogel stability within NeuroFlex and led to significantly improved SC and FB migration compared to the conduit filled with hydrogel alone. This analysis highlights the need for a tailored approach in NGC design, where conduit material characteristics and filling choice are harmonized to create a balanced environment that promotes cellular behaviors essential for nerve regeneration. By examining these material-cell-filling interactions, studies like ours provide critical insights for optimizing regenerative strategies, ultimately guiding the design of NGCs that cater to cellular properties within diverse tissue contexts.

To conclude, our findings revealed why empty nerve conduits consistently underperform by inhibiting SC attachment and migration, underscoring a need for substantial improvements. Fillings such as hydrogel and spider silk significantly enhanced cell attachment and migration, with spider silk emerging as the superior filling. Spider silk not only promoted more effective SC migration but also facilitated directional migration within the hydrogel surrounding the fibers, indicating the potential for structuring fillings in a way that enhances guidance within a three-dimensional matrix. Moreover, this study provides a novel examination of how conduit properties and filling interactions impact cell attachment and migration. The variability in outcomes across collagen and chitosan NGCs emphasizes that conduit morphology —including factors such as porosity, fiber structure, and mechanical properties— can profoundly affect the efficacy of different fillings. This conduit-filling synergy has not been previously explored in such detail and offers new insights for optimizing conduit design.

Our study underscores the critical value of *in vitro* assessments in understanding these interactions before *in vivo* applications, advocating for careful selection and combination of materials in NGCs for improved regenerative outcomes. Based on our findings, we recommend NeuroFlex filled with spider silk fibers, as well as NeuroFlex filled with a combination of spider silk fibers and hydrogel, for future *in vivo* testing. NeuroFlex displayed the most favorable mechanical characteristics and outperformed the other conduits in terms of SC compatibility. While spider silk alone was more effective than the combined hydrogel-fiber condition *in vitro*, the addition of hydrogel may prove advantageous *in vivo* by stabilizing the silk fibers and improving matrix-cell interactions in the tissue context.

### Limitations of the study

Limitations of this study include that the investigation focuses on a specific type of hydrogel (extracellular matrix extract) and *Trichonephila* dragline silk without considering the diverse range of materials available. We have recently uncovered a significant difference in the effect of various spider silks on SC migration^12^ and exploring a broader spectrum of hydrogels or silks might offer more insights into the versatility and potential limitations of different materials in nerve regeneration. Attempts to investigate myelination were hindered as dorsal root ganglion (DRG) neurons failed to adhere to the conduits, precluding these experiments. It might also be of interest to repeat the experiments with NGCs that offer an incorporated inner filling such as the Nerbridge (Toyobo) and NeuraGen 3D Nerve Guide Matrix (Integra).^45^ Lastly, while *in vitro* studies provide detailed insights and controlled environments for initial hypotheses testing, they cannot fully replicate the complex interactions and dynamics within living organisms. Acknowledging this, *in vivo* experiments using the NGCs with the fillings to repair long-distance nerve defects are indeed ongoing and aim to complement our findings by confirming the applicability and effectiveness of filled NGC in a physiological context. This approach ensures that our *in vitro* discoveries are not overshadowed but instead provide a foundational understanding that supports and rationalizes further *in vivo* investigations.

## Resource availability

### Lead contact

Further information and requests for resources should be directed to and will be fulfilled by the lead contact, Flavia Millesi (flavia.millesi@meduniwien.ac.at).

### Materials availability

This study did not generate any new reagents.

### Data and code availability

• All data reported in this paper will be shared by the lead contact upon request.
• This paper does not report original code.
• Any additional information required to reanalyze the data reported in this paper is available from the lead contact upon request.

## Acknowledgments

This project was funded by the Austrian Science Fund: P34750 and P33613. We are immensely grateful for the constant support of the Imaging Core Facility of 10.13039/501100005788Medical University of Vienna. We thank Prof. Ellen Backus at the Department of Physical Chemistry, University of Vienna, for access to the atomic force microscope.

## Author contributions

Conceptualization, F.M.; methodology, F.M., S.M., S.R., Sarah Stadlmayr, G.S., and A.N.; software, F.M., S.M., S.R., Sarah Stadlmayr, and A.N.; validation, F.M.; formal analysis, F.M.; investigation, F.M., S.M., S.R., Sophie Steinwenter, Sarah Stadlmayr, M.H., A.B., L.P., and G.S.; resources, F.M., S.M., S.R., L.S., Sophie Steinwenter, Sarah Stadlmayr, M.H., P.S., and A.B.; writing – original draft, F.M.; writing – review and editing, all authors; visualization, F.M., S.M., S.R., Sarah Stadlmayr, and A.N.; supervision, C.R. and A.N.; project administration, F.M.; funding acquisition, F.M., L.S., A.N., and C.R.

## Declaration of interests

The authors have no conflicts of interest to declare.

## STAR★Methods

### Key resources table

**Table:** 

REAGENT or RESOURCE	SOURCE	IDENTIFIER
**Antibodies**		

NGFR	CellSignaling	RRID: AB_10839265; Cat# 8238S
S100	CellSignaling	RRID: AB_10949319 ; Cat# 9550S
Thy1/CD90	SantaCruz	RRID: AB_630310, Cat# SC-53116
DAPI	ThermoFisher Scientific	Cat# 62248

**Chemicals, peptides, and recombinant proteins**		

MEM α, GlutaMAX™ Supplement, no nucleosides	Gibco	32561–029
Fetal calf serum	PAN Biotech	P40-37500
Penicillin-streptomycin	Gibco	15140122
Sodium pyruvate solution	Gibco	11360–039
4-(2-hydroxyethyl)-1-piperazineethanesulfonic acid buffer solution (HEPES)	Gibco	15630–056
Poly-L-lysine (PLL) hydrobromide	Sigma-Aldrich	P6282
Laminin	R&D Systems	L2020
StemPro™ Accutase	Gibco	A11105-01
Goat Serum	Agilent Technologies	X0907
Forskolin	Merck	F6886
Human Heregulinβ-1	Peprotech	100–03
Human FGF-basic	Peprotech	100-18B
Human PDGF-AA	Peprotech	100-13A
N2-Supplement	Gibco	17502048
Collagenase Type IV	Gibco	17104–019
Dispase II	R&D Systems	D4693

**Critical commercial assays**		

EdU Proliferation Kit	ThermoFisher Scientific	C10338

**Experimental models: Organisms/strains**		

Rats	LEW-Tg(CAG-EGFP)1Ys National BioResource Project, Kyoto, Japan	RRID: RGD_2304291

**Software and algorithms**		

Fiji	ImageJ	RRID: SCR_002285; Version 1.47
Prism software	GraphPad	RRID: SCR_002798; Version 8
R/RStudio	R Project	RRID: SCR_001905; 2024.04.0 + 735
Gwyddion	GNU General Public License	RRID: SCR_015583; Version 2.61

**Other**		

NeuroFlex™	Collagenmatrix	CNCF2025
NeuraGen™ Nerve Guide	Integra	PNG230
Reaxon®	Kerimedical	RD121
Cultrex™ Basement Membrane Extract 3D Cell Culture Matrix	R&D Systems	3445-005-01
μ-slide 8-well chamber slides	ibidi	80826
Cantilevers NSG03	NT-MDT	NSG03

### Experimental model and study participant details

#### Animals

Sciatic nerves from 12 to 20-week-old, male, transgenic inbred ubiquitous GFP^+^ Lewis-Wistar rats (strain LEW-Tg(CAG-EGFP)1Ys, RRID:RGD_2304291*,* National BioResource Project, Kyoto, Japan) were obtained. All rats were housed under standard laboratory conditions with a 12-h light/dark cycle and *ad libitum* access to food and water. All procedures were conducted in accordance with institutional and national ethical guidelines, and complied with the Austrian Animal Testing Law and EU Directive 2010/63/EU. As rats were only used after euthanasia for cell isolation, no ethics approval was necessary. Only male rats were used in this study, and as such, the influence of sex on the results could not be assessed and remains a limitation of the present study.

#### Primary cell culture

SCs and FBs were isolated using a two-step enrichment method described previously.^10^^,^^46^^,^^47^ FBs were cultivated in ∝MEM supplemented with 10% fetal calf serum (FCS), 1% penicillin-streptomycin (P/S), 1% sodium pyruvate solution, and 2.5% 4-(2-hydroxyethyl)-1-piperazineethanesulfonic acid buffer solution (HEPES). SCs were cultured on poly-L-lysine (PLL)/laminin coated dishes using Schwann cell expansion medium^46^^,^^47^ containing ∝MEM supplemented with 5% FCS, 1% P/S, 1% sodium pyruvate solution, 2.5% HEPES, 10 ng/ml Heregulinβ-1 (Peprotech), 10 ng/ml FGF basic (Peprotech), 5 ng/ml PDGF-AA (Peprotech), 2 μM Forskolin (ThermoFisher) and 0.5% N2-Supplement (Gibco). Cryopreserved cells up to passage 4 were used, ensuring purity exceeding 90%.

### Method details

#### Evaluation of material properties

##### Scanning electron and atomic force microscopy

The NGCs underwent thorough analysis using scanning electron microscopy (SEM). The conduits were cut into sections and affixed with conductive double sided carbon tape on an aluminium stub (either as cut circular section or as square piece of the outer or inner wall). A FEI Quanta 250 FEG (Thermo Fisher Scientific, Hillsboro, OR) under high vacuum condition captured micrographs in secondary electron mode at a high tension of 20 kV. The Everhart-Thornley-Detector was used for image recording, offering detailed views of the conduits' structure and surface characteristics. Matrix structure and morphology was evaluated using the ImageJ plug-in TWOMBLI.^48^ Images of four respective positions for conduit wall, inner and outer surface per conduit were quantified. The following parameters were used to evaluate the conduits’ walls: Contrast Saturation, 0.35; Line Width, 5; Max Curvature Window, 50; Minimum Branch Length, 5; Maximum Display High Density Matrix (HDM), 170. The following parameters were used to evaluate the conduits’ surfaces: Contrast Saturation, 0.35; Line Width, 5; Max Curvature Window, 30; Minimum Branch Length, 5; Maximum Display HDM, 170. To calculate the pore size the images were first subjected to a threshold (0–100), then transformed into binary images, dilated and eroded, and, subsequently, analysed (Analyse particles) as described before.^49^

Additionally, atomic force microscopy (AFM) imaging was conducted utilizing a Multimode Atomic Force Microscope (Ntegra Aura NT-MDT). Tapping mode with standard tapping mode cantilevers (NSG03, NT-MDT) with a force constant of around 40 N/m was employed in ambient conditions to capture high-resolution images. Image analysis was performed using Gwyddion software (GNU General Public License. Version 2.61), employing functions remove scars and stepline correction for background correction. Surface area and roughness were quantified using Gwyddion’s statistical quantities tool.

##### Tensile tests and compression resilience tests

To assess mechanical properties, the NGCs were subjected to tensile tests using a universal two-column spindle testing machine from Zwick (MPMS Z1445) equipped with a 500 N load cell. Prior to measurements, the conduits were immersed in distilled water for a minimum of 10 min to replicate wet *in vivo* conditions. The overall sample lengths were 23 mm, measured using a calliper. Toothpicks were inserted for 5 mm at the ends to prevent compression. Samples were pulled at a strain rate of 1 mm/min, and stress-strain curves were recorded for analysis. For each conduit, three replicates were measured. The mean maximum strain as well as the mean breaking stress were calculated from the stress-strain curves.

Moreover, compression resilience tests were conducted similarly to reference.^50^ Wet conduits (1 cm in length) underwent 100 cycles of compression (50% compression rate) between parallel plates. Digital image correlation systems (Q-400 from Dantec Dynamics) equipped with a CCD camera (Manta G-505B from Allied Vision with a Schneider Kreuznach precision lens) captured images after every 10 compressions. For each conduit, three replicates were measured. ImageJ software (1.47) was utilized to measure conduit area changes in each picture, allowing assessment of resilience and deformability properties over multiple cycles.

##### Swelling tests

Conduits were cut longitudinally into 3 mm long pieces and weighed. Consequently, the conduits were submerged in 0.9% (w/v) sodium chloride solution (NaCl) for 10 min at room temperature Afterwards, to elucidate to possible changes after conduit implantation, the conduits were submerged in NaCl for an additional 1 h in the incubator at 37°C and 5% CO_2_ and weighed again. To study the impact of hydrogel on swelling, 9 μL hydrogel was first added on top of the dry conduit pieces and they were weighted. Thereafter, they were incubated in NaCl for 10 min at room temperature. The swelling ratios (SR) were calculating using the dry weights (W_dry_) and wet weight (W_wet_) in the following formula: SR = (W_wet_-W_dry_)/W_dry_∗100.

#### Preparation of conduits

The collagen NGCs NeuraGen® (Integra™) and NeuroFlex™ (Collagen Matrix™) as well as the chitosan NGC Reaxon® (Medovent™) were purchased from the manufacturers. Conduits were adapted to fit μ-slide 8-well chamber slides (ibidi) by being cut longitudinally and reduced to a length of about 7 mm. A 20 μL cell suspension containing either 5.0x10^4^ SCs or 3.0x10^4^ FBs were applied on top of the conduits. The conduits + cells were incubated for 1 h, after which media was added. This made sure that cells attached to the conduit and not on the bottom of the well. Uncoated dishes served as controls.

#### Preparation of conduit filling

For experiments where filling materials were tested, a basement membrane extract hydrogel and spider silk were used as they demonstrated to be potentially effective fillings in past experiments.^11^^,^^13^^,^^17^ An overview of the preparation of conduits and fillings is given in Figure S3.

##### Conduits + hydrogel

A 9 μL drop containing either 5.0x10^4^ SCs or 3.0x10^4^ FBs was applied on top of the conduits and incubated at 37°C and 5% CO2 for 1 h. Subsequently, 10 μL Cultrex® 3D Cell Culture Matrix® Basement Membrane Extract Reduced Growth Factor (Trevigen™) was added on top of the longitudinally cut conduit with the adhered cells. After approximately 5 min, the conduits + hydrogel were submerged in cell culture media.

##### Conduits + spider silk fibres

Spider silk fibres were harvested from adult females of the species *Trichonephila inaurata*. The spiders were fixated on a foam surface and the silk was pulled directly out of the major ampullate gland around a metal frame (Remanium). After being disinfected in ethanol for 10 min and left to dry for at least 30 min, the frames were put into the μ-slide 8-well chamber slides in a way that the silk was in direct contact with the longitudinally cut conduit. A 9 μL drop containing either 5.0x10^4^ SCs or 3.0x10^4^ FBs was then put on top of the spider silk fibres and left in the incubator for 45 min. Consequently, the conduits + fibres were submerged in cell culture media.

##### Conduits + hydrogel + spider silk fibres

For the conduits with both fillings, a 9 μL drop containing either 5.0x10^4^ SCs or 3.0x10^4^ FBs was first put on the fibres on top of the conduit and left in the incubator for 45 min, after which 10 μL hydrogel was added on top of the silk fibres with the cells and the conduit. After approximately 5 min, the conduits + silk + hydrogel were submerged in cell culture media.

#### Immunofluorescence stainings

##### Cell proliferation assay

NGFR and DAPI staining for SC cultures and THY1 and DAPI staining for FB cultures were conducted following procedures from references.^10^^,^^11^^,^^13^^,^^17^^,^^46^^,^^47^ 5-ethynyl-2′deoxyuridine (EdU, Invitrogen) was employed to detect proliferating cells within 2 h. Confocal images were acquired using a LEICA SP8X microscope. EdU^+^/NGFR^+^ SCs and EdU^+^/THY^+^ FBs were counted to determine the number of proliferating cells in the culture. In the Silk+Hydrogel condition, cell proliferating on the silk fibres and in the hydrogel were counted separately.

##### Cell morphology

Using ImageJ, the length (longest line from tip to end of cell) and width (measured perpendicular to length) of 20 SCs and FBs per condition and donor were measured manually and the length/width ratios were calculated. From DAPI staining of SC and FB cultures, nucleus size as well as nucleus roundness were quantified. For this, 50 cells of each condition were automatically evaluated using the function *Analyze particles* in ImageJ.

#### Live cell imaging

Using an Olympus IX83 microscope with a stage-top incubator, live cell imaging of SC and FB cultures was carried out 24 h after seeding similar to our previously published research.^17^ Images were captured every 10 min for 24 h (empty conduits versus uncoated well) and 17 h (empty conduits versus filled conduits), respectively. 20 cells that were trackable throughout the 102 frames were randomly selected for each condition and followed using the Manual Tracking plugin. In the Silk+Hydrogel condition, 40 cells (20 on the silk fibres and 20 in the hydrogel) were analysed. Data were analysed using ImageJ and tracking software for migration characteristics (ibidi Chemotaxis and Migration Tool).

#### Correlation analysis

Pearson correlation analyses were conducted to examine the relationships between conduit material properties and cellular behavior metrics. Specifically, pairwise Pearson correlation coefficients were calculated in R (R Core Team, 2024). between each material property (e.g., tensile strength, compression resistance) and each cellular behavior (e.g., proliferation, elongation, migration speed). Correlation matrices were generated, where each cell represented a correlation coefficient ranging from −1 (perfect negative correlation) to +1 (perfect positive correlation). Heatmaps were then created to visually represent these correlation matrices, with color gradients indicating the strength and direction of the correlation (e.g., positive, negative, or near zero), providing an accessible overview of the relationships across all variables. For the spider charts illustrating how the fillings changed the conduits, data normalization was applied to standardize the values for each cellular behavior metric within each filling type across all conduits. This was done by transforming the data to a scale from −1 to +1, where −1 indicated the minimum value, 1 the maximum, and values in between represented relative performance compared to other conduits for the same filling.

### Quantification and statistical analysis

All quantitative analyses were performed using RStudio (2024.04.0 + 735). Figure legends specify the exact statistical tests used, the definition of n, and the dispersion and central tendency measures (mean ± SD for bar plots and median and interquartile range for boxplots unless otherwise stated). In all analyses, n refers to the mean value per rat, calculated from measurements of individual cells. Statistical comparisons were performed using linear models (ANOVA) with donor as a random effect, followed by Tukey’s post hoc test using the using R-package multcomp 1.4–12.^51^ A significance threshold of *p* < 0.05 was applied throughout. Mean ± SD data were presented (Table S1).

## Supplemental information

Document S1. Figures S1–S3

Table S1. Summary of results in the form of means and standard deviations
