# Supplementary material for: Impact of conduit-filling interactions on the efficacy of fiber and hydrogel fillers in nerve conduits
Source: iScience. 2025 Jul 18;28(8):113150. doi: 10.1016/j.isci.2025.113150 (PMC12357111; doi:10.1016/j.isci.2025.113150)
Supplement: Document S1. Figures S1–S3 [file mmc1.pdf]

iScience, Volume 28

Supplemental information

Impact of conduit-filler interactions on the efficacy of fiber and hydrogel fillers in nerve conduits

Flavia Millesi, Sascha Mero, Sebastian Rihl, Sophie Steinwenter, Sarah Stadlmayr, Anton Borger, Paul Supper, Maximilian Haertinger, Leon Ploszczanski, Gerhard Sinn, Aida Naghilou, Lorenz Semmler, and Christine Radtke

Supplementary Figure 1

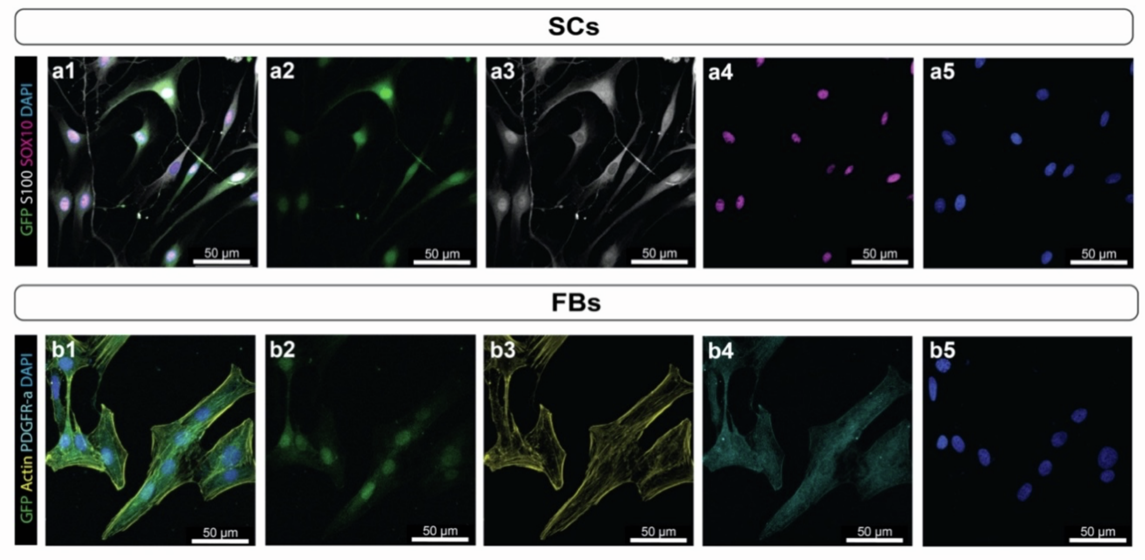

Supplementary Figure 1) Representative confocal micrographs of **a**) SCs expressing 2) GFP and stained for 3) S100, 4) Sox10 and 5) DAPI and **b**) FBs expressing 2) GFP and stained for 3) Actin, 4) PDGFR-a and 5) DAPI.

Supplementary Figure 2

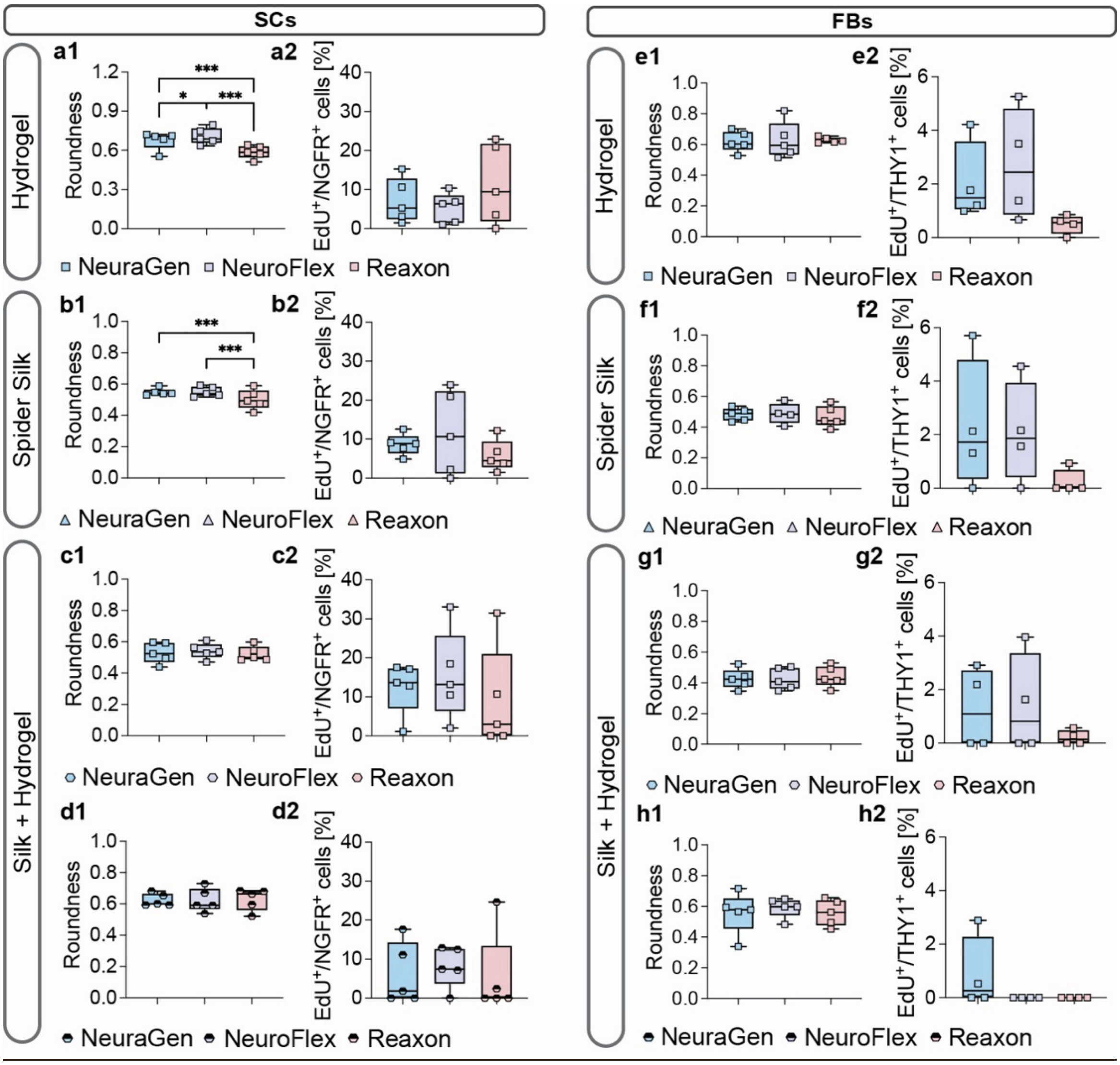

Supplementary Figure 2) The two collagen conduits interact differently with the fillings than the chitosan conduit. a) Mean±SD 1) nuclear roundness, and 2) proliferation rate of SCs in hydrogel (n=5). **b**) Mean±SD 1) nuclear roundness, and 2) proliferation rate of SCs on silk fibres (n=5). **c**) Mean±SD 1) nuclear roundness, and 2) proliferation rate of SCs on silk fibres surrounded by hydrogel (n=5). **d**) Mean±SD 1) nuclear roundness, and 2) proliferation rate of SCs in hydrogel around silk fibres (n=5). **e**) Mean±SD 1) nuclear roundness, and 2) proliferation rate of FBs in hydrogel (n=4-5). **f**) Mean±SD 1) nuclear roundness, and 2) proliferation rate of FBs on silk fibres (n=4-5). **g**) Mean±SD 1) nuclear roundness, and 2) proliferation rate of FBs on silk fibres surrounding hydrogel (n=4-5). **h**) Mean±SD 1) nuclear roundness, and 2) proliferation rate of FBs in hydrogel around silk fibres (n=4-5). * p-value < 0.05, ** p- value < 0.01, *** p-value < 0.001, two-way ANOVA.

Supplementary Figure 3

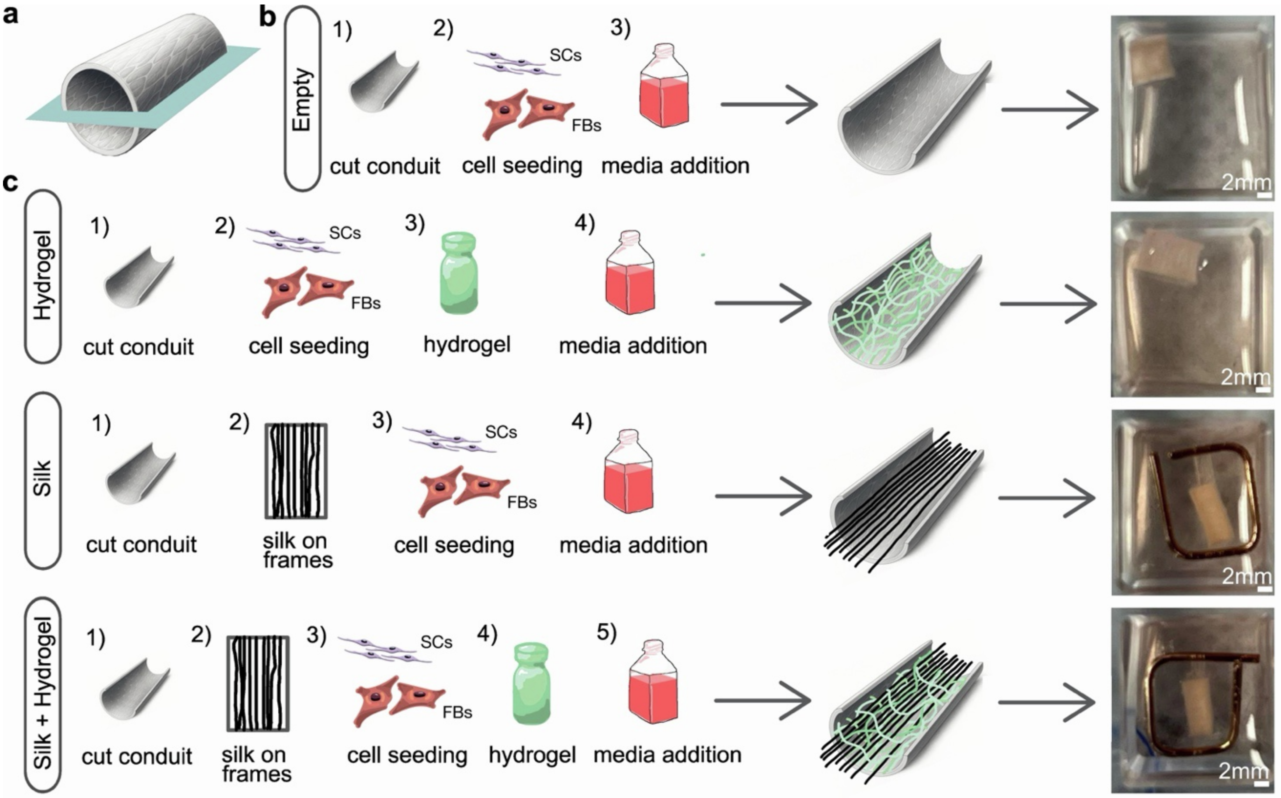

Supplementary Figure 3) Preparation of conduits. a) Conduits were cut longitudinally. b) For the empty conduits, cells were seeded on top of the cut conduit and media was added after 45 minutes. c) Preparation of filled conduits; For the hydrogel-filled conduit, cells were seeded on top of the cut conduit and 10 µl hydrogel were added on top after 45 minutes. Consequently, conduits + cells + hydrogel were submerged in media. For the silk-filled conduits, the conduits were placed in-between silk fibres reeled around a metal frame, allowing the fibres to have direct contact with the conduits. Cells were seeded on top of the silk fibres on the conduit and incubated for 45 minutes, after which media was added. Lastly, for the conduits filled with hydrogel and spider silk, the cells were first seeded on the fibres on top of the conduits and incubated for 45 minutes. Then, a 10 µl drop of hydrogel was placed on top and after 5 minutes, the conduits + silk + cells + hydrogel were submerged in media.
